# A novel dengue virus detection method that couples DNAzyme and gold nanoparticle approaches

**DOI:** 10.1186/1743-422X-10-201

**Published:** 2013-06-28

**Authors:** James R Carter, Velmurugan Balaraman, Cheryl A Kucharski, Tresa S Fraser, Malcolm J Fraser

**Affiliations:** 1 Department of Biological Sciences, Eck Institute of Global Health, University of Notre Dame, Notre Dame, IN 46556, USA

**Keywords:** Dengue, Flavivirus, DNAzyme, Nanoparticles, Gold, Arbovirus, Detection

## Abstract

**Background:**

Recent epidemics of dengue viruses (DENV) coupled with new outbreaks on the horizon have renewed the demand for novel detection methods that have the ability to identify this viral pathogen prior to the manifestation of symptoms. The ability to detect DENV in a timely manner is essential for rapid recovery from disease symptoms. A modified lab-derived 10-23 DNAzyme tethered to gold nanoparticles provides a powerful tool for the detection of viruses, such as DENV.

**Results:**

We examined the effectiveness of coupling DNAzyme (DDZ) activation to the salt-induced aggregation of gold nanoparticles (AuNP) to detect dengue virus (DENV) progeny in mosquito cells. A DNAzyme was designed to recognize the 5’ cyclization sequence (5’ CS) that is conserved among all DENV, and conjugated to AuNPs. DDZ-AuNP has demonstrated the ability to detect the genomic RNA of our model dengue strain, DENV-2 NGC, isolated from infected *Aedes albopictus* C6/36 cells. These targeting events lead to the rapid aggregation of AuNPs, resulting in a red to clear color transition of the reaction mixes, and thus positive detection of the DENV RNA genome. The inclusion of SDS in the reaction mixture permitted the detection of DENV directly from cell culture supernatants without additional sample processing. Specificity assays demonstrated detection is DENV-specific, while sensitivity assays confirm detection at levels of 1 × 10^1^ TCID50 units. These results demonstrate DDZ-AuNP effectively detects DENV genomes in a sequence specific manner and at concentrations that are practical for field use.

**Conclusions:**

We have developed an effective detection assay using DNAzyme catalysis coupled with AuNP aggregation for the detection of DENV genomes in a sequence specific manner. Full development of our novel DDZ-AuNP detection method will provide a practical, rapid, and low cost alternative for the detection of DENV in mosquito cells and tissues, and possibly infected patient serum, in a matter of minutes with little to no specialized training required.

## Background

Dengue viruses (DENV), members of the Flavivirus family of viruses, cause periodic explosive epidemics in many tropical and sub-tropical countries leading to approximately 50-100 million infections per year [1-5]. Approximately 500,000 of these are severe cases requiring hospitalization with a 2.5% fatality rate, most of which are children [1-5]. Approximately half the world’s population remains at risk for DENV infection making this pathogen one of the most dangerous viruses in the world [6]. In 2010 there were 1.6 million cases of Dengue in the Americas alone, of which 49,000 were severe cases [1]. Recent domestic outbreaks have occurred in the Hawaiian Islands in 2001 [7], Brownsville, Texas in 2005 [8], the Florida Keys in 2010 [9] and other parts of southern Florida including Miami-Dade in 2011 [10,11]. Furthermore, devastating outbreaks continue to occur in Puerto Rico [10,12,13], Brazil [14], and Pakistan [15], to name a few.

DENV are maintained in a cycle that involves humans and the globally disseminated *Aedes aegypti* mosquito [16]. Infection with one of four antigenically distinct, but genetically related DENV serotypes (designated DENV-1, -2, -3, and -4) can result in dengue fever (DF) and/or potentially fatal dengue hemorrhagic fever (DHF) [17]. These disease states are characterized by high fever, often with enlargement of the liver, and in severe cases circulatory and respiratory failure [3]. While DF and DHF are endemic to tropical and subtropical regions of the world, collapse of effective vector control programs, rapid dispersal of viruses due to ease of global travel, and migration of humans from tropical to non-tropical regions has resulted in DENV outbreaks in regions that were once non-endemic to these viral pathogens.

The ability to detect DENV in a timely manner is essential to rapid recovery from disease symptoms. Currently, detection of mosquito-borne viruses in infected persons is limited to plaque assays, antigen detection assays (e.g. NS1 antigen detection), or quantitation of viral production through PCR-based methods [18-20]. These assays are currently referred to as the “gold standards” for DENV detection [21-24]. More relevant to our research, current testing of mosquito populations for arboviruses in general, but more specifically dengue viruses, has been limited to RT-PCR of mosquito pools (25-100 insects) [25-27].

The approaches mentioned above are limited by a number of pitfalls including low-throughput, labor-intensiveness, low stability of assay components at or above room temperature, and lack of portability. The requirement for specialized training and equipment and the time consuming nature of these assays limits their widespread utility for virus detection. These limitations compromise rapid diagnosis of viral infections. Additionally, these methods are not easily adapted to field environments where reliable and effective detection methods are needed. Rapid, low-tech virus detection methods that require no specialized training or education are sorely needed to provide remote areas of the world the ability to detect highly pathogenic viruses for both clinical diagnosis and epidemiological surveillance.

**Figure 1:**
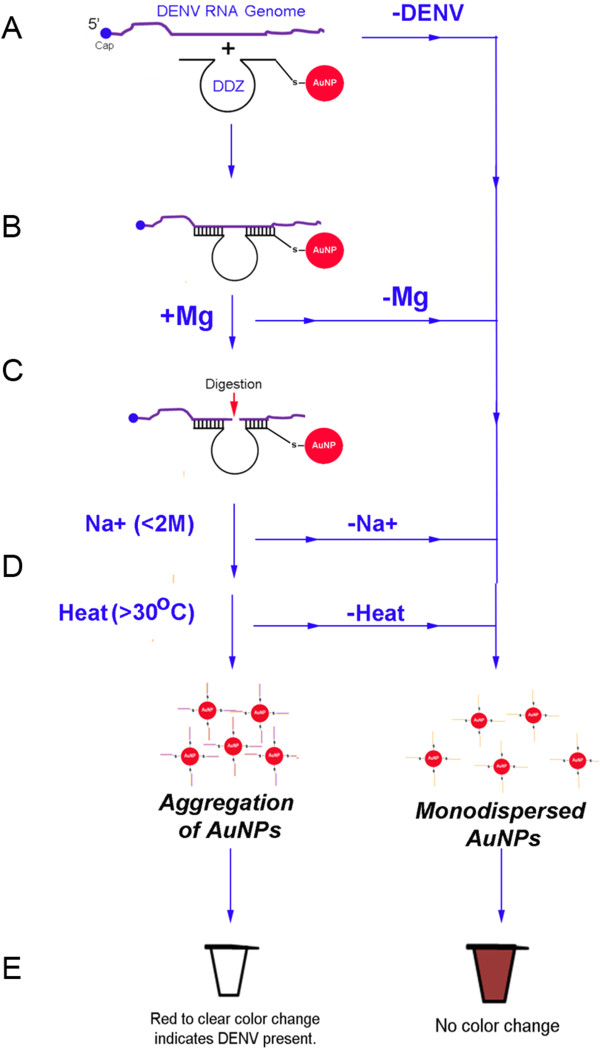
**Overview of the DDZ-AuNP assay for dengue virus detection**. Schematic of the DENV detection system using DENV-specific DNAzyme (DDZ) catalysis coupled with gold nanoparticle (AuNP) aggregation. AuNPs are conjugated with the sulfide-linked anti-DENV DNAzyme, DDZ, which is complimentary to the DENV RNA genome (shown in orange). Black vertical lines indicate complimentary base pairing between DDZ and the target RNA. In the presence of DENV RNA **(A)**, the 5’ and 3’ arms of the anti-DENV DNAzyme, DDZ, bind to the 3’ and 5’ ends of the targeted 5’-3’ CS region, respectively **(B)**. When Mg2+ and heat (37°C) are introduced DDZ digests the viral RNA **(C)**. This digestion results in deshielding of the AuNP, leading to aggregation of these AuNPs in the presence of NaCl and heat **(D)** allowing a rapid and visually detectable red to clear color transition **(E)**. This color transition signifies the successful detection of DENV, and can be quantified by UV/Vis spectrophotometry at 520 nm AuNPs = red, tethered DNA probe = orange, DENV genome = purple.

In this report we describe the development and initial validation of a colorimetric DENV detection method that couples the RNA targeting ability of a DENV-specific DNAzyme (DDZ) with the aggregation properties of oligonucleotide-tethered, noncrosslinking gold nanoparticles (AuNPs). Our innovative DENV detection system, called DDZ-AuNP (Figure 1), should be an invaluable tool for the detection of DENV since it solves many of the limitations of current virus detection assays. This assay and subsequent analysis is cost effective, simple to perform, and the assay components are highly stable at temperatures above 30°C enabling easy storage at room temperature. The use of DNAzymes in the assay increases the specificity and versatility of detection permitting the design and incorporation of additional virus or strain-specific DNAzymes and probes.

Full development of this detection assay would greatly enhance virus diagnostics and epidemiology by providing an assay that is more rapid, easier to use, has greater portability, and is more cost effective than current DENV detection methods.

## Results

### Overview of the colorimetric detection of DENV by DNAzyme activity coupled with noncrosslinking AuNP aggregation (DDZ-AuNP)

The dengue virus detection method described below (see Figure 1) is based upon RNA aptazyme-mediated detection of small molecules, such as theophylline [28,29]. The central limitation of using RNAzymes and aptazymes for virus detection is the inherent instability these catalytic RNAs, increasing the attractiveness of DNAzymes in detection assays.

The colorimetric detection of DENV by DDZ-AuNP can be divided into three phases: targeting/cleavage of the DENV RNA genome by DDZ, activation of AuNPs, and aggregation of AuNPs and detection (Figure 1). In the presence of DENV the 5’ and 3’ arms of DDZ bind to the 3’ and 5’ ends of a fully conserved segment that includes the 5’-3’ CS region, respectively, and in the presence of Mg^2+^ and heat (37°C) the viral RNA is cleaved at G149. This cleavage results in deshielding and aggregation of the AuNPs in the presence of NaCl and heat [30-32], causing a visually detectable red to clear color transition [31,32] that can be quantified by UV/Vis spectrophotometry at 520 nm [31,32]. If DENV are not present in the sample, the DNAzyme -tethered AuNPs remain in a dispersed state and no color loss should occur [31,32]. Likewise, if any of the essential components such as magnesium or sodium are not present in the reaction mixture, no aggregation is possible.

### Design and *in vitro* cleavage assessment of the DENV detection system DDZ-AuNP

**Figure 2:**
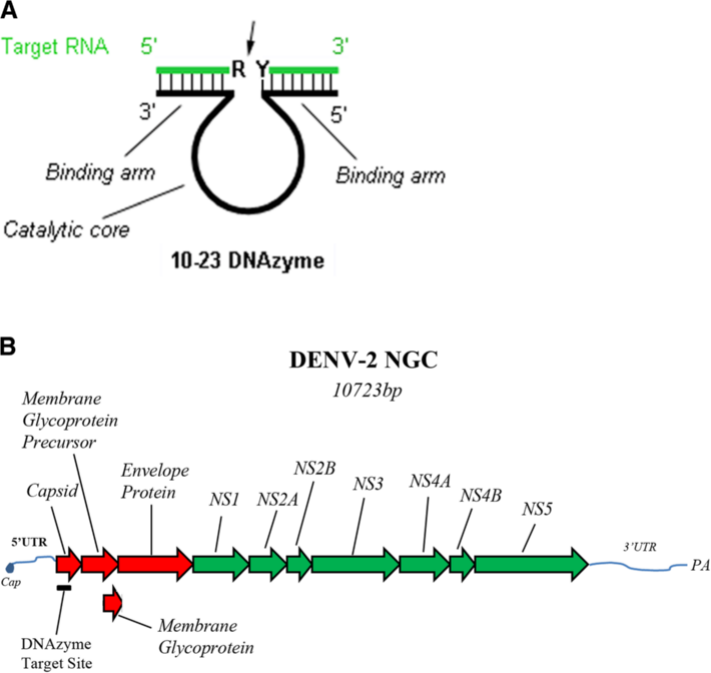
**Anatomy of the 10-23 anti-DENV DNAzyme (DDZ) and Schematic Diagram of the DENV genome. A)** DDZ was designed and produced as previously described [36,85] and in Materials and Methods. R = Purine. Y = Pyrimidine. The target RNA is in green with the anti DENV DNAzyme 10-23 shown in black. The 5’ and 3’ ends of target RNA and DNAzymes are as indicated. Thin black vertical lines show complimentary base pairing. Arrows indicate cleavage site of the target RNA. **B)** A representation of the 10,723 base DENV-2 NGC GC capped and polyadenylated genome is shown to illustrate the position of the region targeted by the anti-DENV DNAzyme, DDZ. Non structural (NS) genes 1 through 5 are shown in green. Structural genes encoding capsid, membrane glycoprotein precursor, and envelope proteins are shown in red. UTR = untranslated region, PA = polyadenylation.

DNAzymes are lab-derived, auto catalytic DNAs consisting of three intimately connected domains (Figure 2A): A catalytic core that is activated by binding a cofactor (eg. Pb^2+^ or Mg^2+^) [33-36] (though a few DNAzymes do not require a cofactor) [37], and 5’ and 3’ binding arms that bind to the 3’ and 5’ regions of the target sequence, respectively. DNAzymes have demonstrated impressive sensitivity in detecting metal ions or RNA [38-45].

The 10-23 DNAzyme is capable of cleaving RNA with high sequence specificity at target sites containing purine-pyrimidine (R-Y) junctions [46]. We chose the 10-23 DNAzyme for use in our DENV detection system because this DNAzyme is less dependent on secondary structure formation for its activity than other DNAzymes [47] and would be expected to perform better in our *in vitro* assays where biomolecular folding would be quite variable. The design of the anti-DENV 10-23 DNAzyme, DDZ-M (Figure 2A), was based on a 10-23 DNAzyme clone that was discovered through SELEX (Systematic Evolution of Ligands by Exponential Enrichment). We designed the 5’ and 3’ arms to target the highly conserved region that includes the 5’-3’ cyclization sequence (CS) that is present in all DENV serotypes, and is required for replication of genomic RNA (Figure 2A) [48,49].

Gold nanoparticles (AuNPs) ranging from 15 nm to 100 nm in diameter have been used in a number of detection assays [30,50,51]. We chose to conjugate DDZ to 15 nm AuNPs since fewer copies of single-stranded DNA are required to cover the surface of a 15 nm AuNP than any AuNP of larger size [52], and interaction of only 7.5% of DNAs conjugated to the 15 nm AuNPs with the substrate RNA is required to initiate aggregation of the AuNPs [30].

AuNP-conjugated DDZs were analyzed for their ability to cleave the DENV-2 NGC RNA *in vitro*. DENV-2 NGC viral RNAs were isolated from infected *Ae. albopictus* C6/36 cells, and incubated in a buffered solution containing 1 × 10^5^ DDZ-M-tethered AuNPs/mL for 30 minutes at 37°C. Digestion products were then amplified by RT-PCR using heterologous and hexamer primers designed to aid in the amplification of DDZ digestion products.

**Figure 3:**
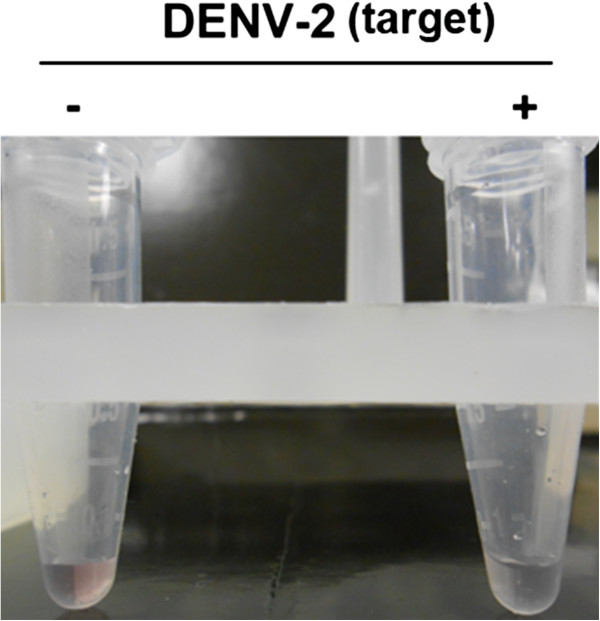
**In vitro DDZ activity assay.** DDZ-M-AuNPs (1 × 10^5^ particles/mL) were placed in a buffered solution containing 10 mM MgCl_2_, DENV-2 NGC RNAs (0.6 μM) isolated from *Ae. albopictus* C6/36. Following incubation at 37°C for 30 minutes, RT-PCR was performed as described in Materials and Methods to amplify digestion products. Fragments were then separated on a 1.75% TAE agarose gel in the presence of ethidium bromide and photographed under UV light. Arrows indicate digestion products of approximately 150 and 350 bases and the primer dimer, respectively. Results demonstrate DDZ-M digestion of full length DENV-2 genome in spite of AuNP conjugation. DDZ = anti-dengue virus DNAzyme, DDZin = inactive anti-dengue virus DNAzyme.

Successful digestion of the DENV-2 NGC RNA genome by DDZ-M was demonstrated by the positive detection of 2 fragments of approximately 150 and 350 bases in size, indicative of DDZ-M catalysis (Figure 3). DNAzyme catalytic activity against the DENV-2 RNA genome was validated by the inclusion of an inactive DNAzyme negative control, DDZin-M, that was created by inverting the catalytic domain which has been previously shown to render the DNAzyme catalytically inactive [41]. As expected, the catalytically inactive DDZin-M did not digest the DENV-2 NGC genome due to this alteration in the catalytic domain.

### Addition of an artificial DENV-2 RNA target initiates aggregation of DDZ tethered AuNPs

As an initial test of the utility of our colorimetric detection method a synthetic target was designed and synthesized that corresponds to the 5’ 170 bases of the DENV-2 NGC genome. This stretch of nucleotides included the highly conserved 5’ CS domain and the initial 74 bases of the capsid gene [49,53,54].

**Figure 4:**
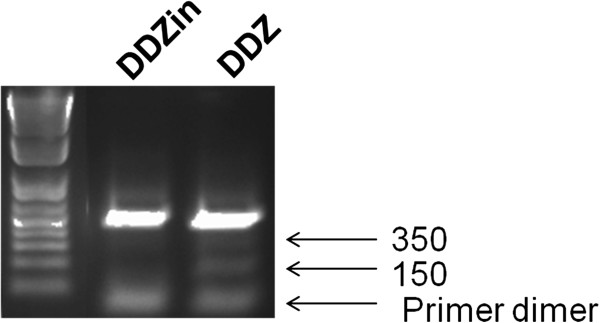
**Colorimetric DDZ-AuNP detection of a synthetic DENV-2 NGC RNA target.** A synthetic stretch of ribonucleotides corresponding to the 5’ 170 bases of the DENV-2 RNA genome was added to a buffered mixture containing 10 mM MgCl_2_, 1 × 10^5^ DDZ-M-AuNP particles/mL, and 1.0 M NaCl. Samples were incubated at 37°C for 5 minutes and photographs were taken. Control samples were treated the same as experimental except 50 mM Tris–HCl was added in lieu of the synthetic DENV-2 RNA. Aggregation of DDZ-M tethered AuNPs only occurred in the presence of synthetic DENV RNA. Results indicate that DDZ-AuNPs have the ability to detect DENV. DDZ = anti-DENV DNAzyme.

Synthetic target (7.5nM) was added to a buffered mixture containing 1 × 10^5^ DDZ-AuNPs/mL, 10 mM MgCl_2_ and1.0 M NaCl (Figure 4) as previously described [31,32]. The control mix contained the same components except 50 mM Tris HCl was substituted for the synthetic DENV-2 target. Reaction mixes were incubated at 37°C to initiate the detection reaction. Aggregation of the DDZ-M--tethered AuNPs, observed by a red to clear color transition, was evident within the first 5 minutes of incubation. This aggregation event occurred only in the presence of the synthetic DENV-2 RNA, and therefore demonstrated a positive test for the presence of DENV-2 RNA.

### Optimization of NaCl concentration

Sodium, in the form of NaCl, is an essential component of AuNP colorimetric detection assays because this monovalent salt drives aggregation of oligonucleotide-conjugated AuNPs [30,55-57] following the interaction of the AuNP conjugated probes with complimentary oligonucleotide targets [58,59]. However, NaCl concentrations greater than 2 M can cause instability of conjugated AuNPs [30]. Furthermore, published reports indicate that NaCl concentrations for effective AuNP aggregation can vary from 1.0 M to 1.5 M [51,60]. In light of these prior observations, we evaluated the optimal NaCl concentration necessary to initiate aggregation of DDZ-M-AuNP following interaction with the DENV-2 genome.

**Figure 5:**
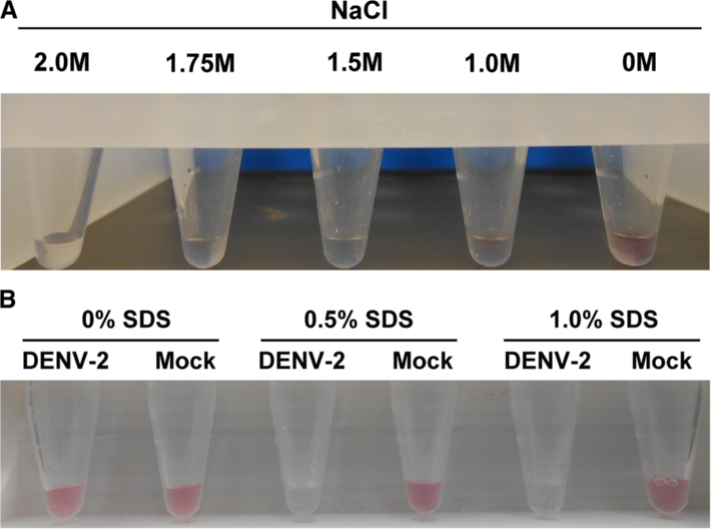
**Determination of optimal NaCl and SDS concentrations. A)** The optimal concentration of sodium, in the form of NaCl, for aggregation of DDZ-M-AuNP following interaction with the DENV-2 RNA genome was determined. DENV -2 NGC strain genomic RNAs were isolated as described in Materials and Methods and 0.6 μM was incubated in a reaction mix containing DDZ-M-AuNP (1 × 10^5^ particles/mL), 10 mM MgCl_2_, and increasing concentrations of NaCl (0 to 2 M) for 30 minutes at 37°C. A representative photograph of the reaction tubes is shown. The concentration of NaCl is indicated above each reaction tube. A full red to clear color transition indicates the optimum detection of the DENV-2 NGC genome. 1.5 M NaCl was determined to be the minimal optimum concentration of NaCl to use in our DENV detection reactions. **B)** The optimal concentration of SDS was determined in the presence of DENV-2 NGC virions. C6/36 cells were infected with DENV-2-NGC (MOI = 0.1). At 6dpi, 10 μl of cell supernatants containing 1 × 10^6^ DENV-2 NGC/mL, as determined by TCID_50_-IFA, were added to a reaction mix containing 10 mM MgCl_2_, 1 × 10^5^ DDZ-M-AuNP particles/mL, 1.5 M NaCl, and 0% (w/v) to 1% (w/v) SDS detergent. Samples were incubated at 37°C for 30 minutes and photographs were taken. Results demonstrate that the DDZ-M-AuNP colorimetric method for DENV detection occurs optimally in 0.5% SDS. The percent SDS used is indicated above each eppendorff tube. SDS = sodium dodecyl sulfate.

DENV genomic RNAs (~0.6 μM), isolated from infected C6/36 cell supernatants, were added to a buffered reaction mixture containing DDZ-M–AuNP (~1 × 10^5^ particles/mL), 10 mM MgCl_2_ and NaCl at concentrations ranging from 0 M to 2 M (Figure 5A). Samples were incubated at 37°C for 30 min. A red to clear color transition confirming optimal detection of the DENV genome was observed in as little as 5 minutes in the presence of 1.5 M NaCl. The 0 M NaCl control provided confirmation that the red to clear color transitions observed were not the result of destabilization of aggregates from DNAzyme activity against the AuNPs, nor were they caused by non-specific interaction of the DNAzymes with cell derived oligonucleotides. Our results also demonstrate the high stability and utility of our DDZ -AuNP assay at temperatures greater than 30°C, a critical criterion for any DENV detection assay [61].

### Determination of the optimal SDS concentration for colorimetric DDZ-AuNP detection of DENV

Our DDZ-AuNP assay system demonstrated utility in detecting purified DENV-2 RNAs. However, to improve this assay for field use we needed a protocol that has speed, efficacy, and simplicity in detecting DENV RNA directly from virions. Liberating the DENV RNA genome from virion particles using a low cost, non-toxic RNA extraction reagent that is stable in the reaction buffer and does not interfere with the assay would be ideal. Sodium dodecyl sulfate (SDS) is an effective non-ionic detergent for lysing virus particles [62]. SDS may be considered an ideal component for our colorimetric detection assays because it is non-toxic, stable in the reaction buffer, and does not require additional manipulation during lysis.

The optimal concentration of SDS was determined by adding cellular supernatants containing 1 × 10^6^ DENV-2/mL to buffered reaction mixes containing DDZ -tethered AuNPs (DDZ-AuNP), 10 mM MgCl_2_ and SDS at concentrations of 0% (w/v), 0.5% (w/v) or 1.0% (w/v) (Figure 5B). Detection of DENV-2 NGC RNAs from cell culture fluid was not possible in the absence of SDS following incubation at 37°C for 30 min. Similarly, controls involving mock infected cell supernatants with or without SDS showed no red to clear color change distinctive of AuNP aggregation. However, infected cell culture supernatants displayed positive detection in as little as 5 minutes, and only in the presence of SDS and DENV-2 NGC. AuNP aggregation in the presence of 0.5% SDS and absence of DENV-2 virus particles was undetectable.

### Measurement of Mg^2+^ resistance of oligonucleotide-tethered AuNPs

**Figure 6:**
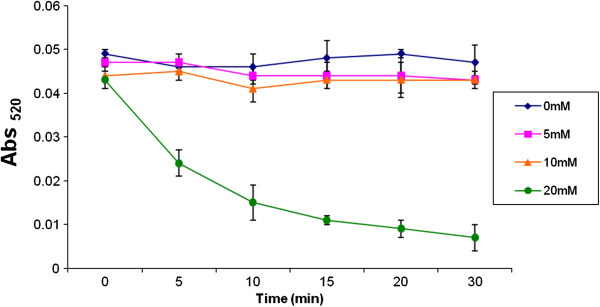
**Assessing Mg**^**2+ **^**resistance of DDZ-tethered AuNPs.** DDZ-M tethered AuNPs were incubated with increasing concentrations of MgCl_2_ (0 to 20 mM) as described in Methods. Following a 30 minute incubation period at room temperature (~25°C), UV/Vis spectrophotometry and photography were performed. These results demonstrate that DNAzyme conjugated AuNP aggregation is not driven by 10 mM MgCl_2_, which is used in all detection assays described in this report.

Since DDZ is activated by 10 mM MgCl_2_, we needed to confirm that the positive detection of DENV-2 was due to specific recognition of the viral genome by DDZ-M-AuNP and not the result of a false positive from Mg^2+^ ion destabilization of DDZ-AuNPs [30,55-57]. The stability of DDZ-M-AuNP was tested against increasing concentrations of MgCl_2_ (0 mM to 20 mM) at room temperature every 5 minutes for up to 30 minutes (Figure 6), and absorbencies were measured with a NanoDrop spectrophotometer at 520 nm. As expected, concentrations equal to or less than 10 mM MgCl_2_ did not display a detectable effect on the stability of the oligonucleotides-tethered AuNPs as evidenced by a lack of aggregation and absorbance, while those above 10 mM resulted in rapid instability of DDZ-AuNP, leading to aggregation of the nanoparticles as evidenced by the rapid decrease in absorbance.

### Specificity of DDZ-AuNP for DENV

**Figure 7:**
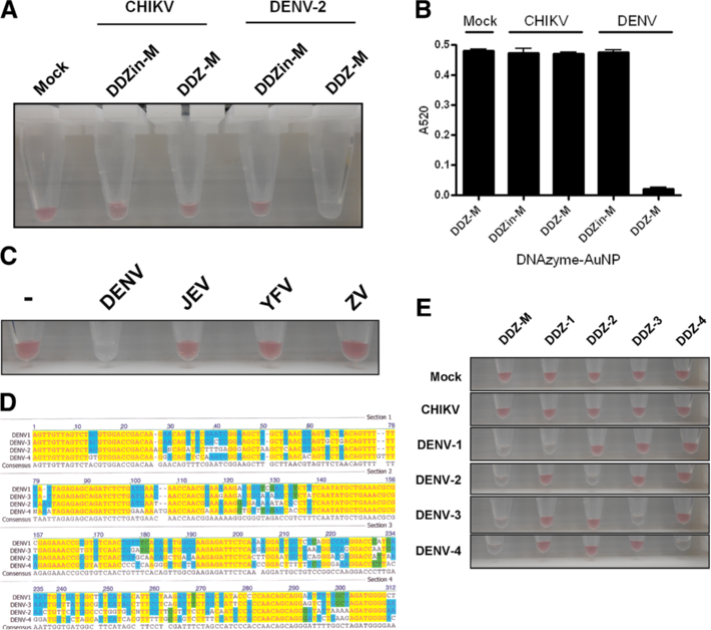
**Assessment of DDZ-AuNP specificity. A)** DENV-2 and CHIKV (1 × 106/mL each) were placed in a buffered solution containing 10 mM MgCl2, 1 × 105 DDZ-AuNP particles/mL, 1.5 M NaCl, and 0.5% (w/v) SDS. Eppendorf tubes containing these mixes were incubated at 37°C for 30 minutes and photographed. CHIKV = chikungunya virus. DENV-2 = dengue virus serotype 2, DDZ-M = anti-dengue virus DNAzyme, DDZin-M = inactive anti-dengue virus DNAzyme. **B)** Analysis of DENV detection by UV/Vis Spectrophotometry. Samples were assembled as was performed for in A, mixed, incubated at 37°C for 5 minutes, and spectrophotometric analysis was performed using the ND-1000 spectrophotometer. **C)** Detection of DENV by DDZ-M-AuNP in comparison to several other flaviviruses. The specificity of our DDZ-M-AuNP device to detect DENV and not other fellow flavivirus members YFV, JEV or ZV was determined as described earlier (see Figure 4). **D)** An alignment was performed on consensus sequences of each of the four DENV serotypes to determine the most optimal regions for the design of serotype specific DDZ-AuNP devices by determining the region of least conservation one serotype has with the other DENV serotypes. **E)** Colorimetric serotype-specific detection of DENV. Cell culture supernatants from C6/36 cells mock infected (Mock), or from cells infected with either DENV serotypes 1 through 4 or CHIKV (1 × 106/mL each) were placed in buffered solutions containing the necessary cofactors and AuNPs tethered with our all purpose DENV serotype specific DNAzyme, DDZ-M, or one of the serotype-specific DDZs (designated DDZ-1 through-4) designed to specifically target the corresponding DENV serotype (Table 1). Eppendorf tubes containing these mixes were incubated at 37°C for 5 minutes and photographed.

Because Chikungunya virus (CHIKV) and DENV co-infections have become more prevalent in South Asia and Africa [63-65], we tested our DDZ-AuNP detection method for its specificity for DENV in the presence of CHIKV (Figure 7A). Cellular supernatants containing 1 × 10^6^ DENV-2 or 1 × 10^6^ CHIKV/mL, as determined by TCID_50_-IFA [53,66] (1 TCID50 unit = 0.7 virus plaque forming units (pfu)) were added to a buffered reaction mixture containing 1 × 10^5^ DDZ-M or DDZin-Mtethered AuNP/mL, 10 mM MgCl_2_, 1.5 M NaCl and 0.5% (w/v) SDS. As expected when gold nanoparticles tethered with DDZ-M DNAzymes were incubated with either mock infected or CHIKV infected cell supernatants, AuNP aggregation did not occur. Furthermore, the substitution of DDZ-M-AuNP with the negative control DDZin-M-AuNPs also resulted in negative detection of DENV. However, positive detection of DENV-2 NGC was observed in as little as 5 minutes, when DDZ-M-AuNP was incubated with DENV in fected C6/36 cell derived supernatants. These results demonstrated DDZ-M-AuNP could specifically detect DENV in these mixed virus samples.

An important feature of using gold nanoparticles in colorimetric detection schemes is that the aggregation of AuNPs can be detected by UV/Vis spectroscopy. Since the absorption maximum of the 15 nm AuNPs used in this detection method is 520 nm, a decrease in absorbance at 520 nm can also be used to detect and quantitate aggregation. This was tested using reaction mixtures containing cell culture supernatants from DENV infected cells (Figure 7B). UV/Vis spectrophotometric analysis at A520 showed a decrease in absorbance when DDZ-M-AuNP positively detected DENV-2, suggesting the ability to quantitate these aggregation events. Mock or CHIKV infected cell culture fluids, or AuNPs tethered with the catalytically inactive DDZin-M do not elicit a detectible change in absorbance. These results show that our colorimetric DDZ-AuNP method for DENV detection possesses utility in a UV/Vis spectrophotometric platform.

DENV shares similar symptoms with other closely related mosquito-borne flaviviruses, such as Yellow Fever (YFV) [67,68], Japanese Encephalitis (JEV) [69], and Zika (ZV) [70,71] viruses. These viruses also co-circulate with DENV and are often misdiagnosed as dengue. Therefore, a DENV detection method must demonstrate the ability to distinguish DENV, from other mosquito-borne flaviviruses. Although the 5’-3’ CS domains are largely (but not fully) conserved among flaviviruses, the entire DDZ-M binding site is not conserved among all these flaviviruses as demonstrated by a sequence alignment of our DDZ-M binding site with corresponding regions in YFV, JEV, and ZV viruses. We also performed a experimental analysis of our DDZ-M-AuNP assay to verify its ability to distinguish DENV over other flaviviruses. Separate reaction mixtures were assembled as previously described (see Figure 4), except that artificial RNA substrates comprised of the 5’ 220 nucleotides of the YFV, JEV, ZV, and DENV genomes were used as targets (Figure 7C). This stretch of nucleotides included the highly conserved 5’ CS domain and the initial 74 bases of the capsid genes of each flavivirus. Aggregation of the DDZ-M-tethered AuNPs was evident only in the presence of the artificial DENV-2 RNA substrates and not YFV, JEV, or ZV. AuNPs tethered with the catalytically inactive DDZin-M did not aggregate in the presence of any flavivirus RNA substrate tested illustrating that mere binding of an RNA substrate is not enough to elicit an aggregation response by AuNPs. These results further validated the specificity of our DENV detection method.

Lastly, to be effective in epidemiological surveillance efforts, a DENV detection method must demonstrate the ability to detect each serotype independently of the other. An alignment of all four known DENV serotypes was performed to determine the ideal target sites for the design of serotype specific DNAzymes (Figure 7D) and appropriate targeting sequences were assembled (Table 1). Serotype- DNAzyme-tethered AuNPs were tested for their ability to detect viral genomic RNAs of DENV serotypes 1 through 4 (Figure 7E). AuNPs-tethered with either a serotype-specific DDZ or the multiple serotype detecting DDZ-M were placed in separate mixtures containing the DENV serotype indicated, 0.1% SDS to lyse virus particles, and 1.5 M NaCl (Figure 7E). Mixes were incubated at 37°C for 5 min.

**Table 1:** Summary of active and negative control DDZ-AuNP devices

**Devices**	**Designed to detect**	**Serotype detected**			
		**DENV-1**	**DENV-2**	**DENV-3**	**DENV-4**
DDZ-M-AuNP	All Serotypes	+	+	+	+
DDZ-1-AuNP	DENV-1	+	-	-	-
DDZ-2-AuNP	DENV-2	-	+	-	-
DDZ-3-AuNP	DENV-3	-	-	+	-
DDZ-4-AuNP	DENV-4	-	-	-	+
*Negative Control*					
DDZin-M-AuNP	None	-	-	-	-

Left column lists the active (DDZ-M-AuNP and DDZ-1-AuNP through DDZ-4-AuNP) and inactive negative control (DDZin-M-AuNP) devices used in this report. The second column lists the serotype each device was designed to detect. Lastly, the right column lists the summarized results of the DENV detection devices and the negative control DDZin-M-AuNP. See Methods for description of DNAzyme design.

The DENV-1 serotype-specific DDZ-1-AuNP positively detected the DENV-1 serotype as signified by a distinctive red to clear/colorless color transition. As expected, DDZ-1-AuNP did not detect DENV-2, -3, or -4, illustrating the serotype-specific nature of this approach (Figure 7E). Likewise, each of the other serotype specific DNAzyme tethered AuNPs detected only the corresponding DENV serotype (Figure 7E and Table 1). These results demonstrated a DENV detection method that couples DNAzyme activity with AuNP aggregation to identify DENV in a serotype-specific manner. Cell culture supernatants containing the negative control CHIKV were added in lieu of DENV to further demonstrate the specificity of the serotype specific AuNPs and overall feasibility of our DENV detection assay. As expected, neither mock infected nor CHIKV infected cell culture supernatants yielded the red to clear color transition typically observed for the positive detection of DENV, showing our conjugated AuNPs were not influenced by cell or CHIKV derived oligonucleotides.

### The limits of DDZ-AuNP colorimetric detection of DENV-2

**Figure 8:**
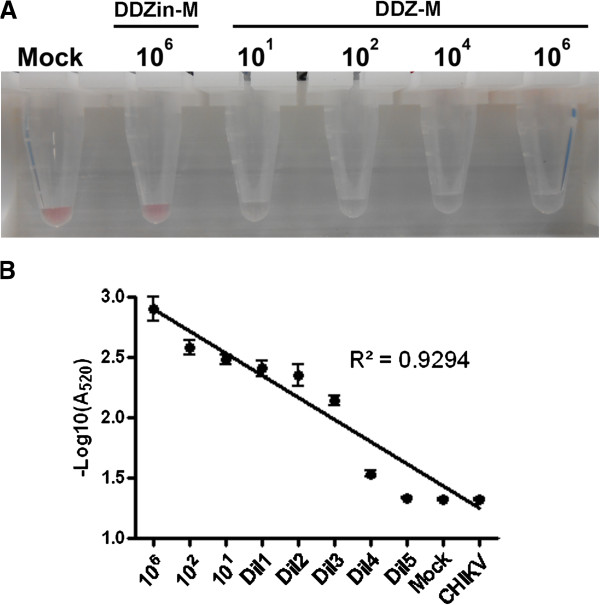
**Analysis of DDZ-AuNP sensitivity. A)** DDZ-M-AuNP colorimetric assays were performed on DENV-2 NGC titers of 1 × 10^1^, 1 × 10^2^, 1 × 10^4^, and 1 × 10^6^ to determine the limits of detection. Samples were assembled as described in Methods, incubated at 37°C for 30 minutes and photographed. Results show that the DDZ-M-AuNP colorimetric assay is capable of detecting DENV-2 at a titer as low as 1 × 10^1^. DDZ-M = anti-dengue virus DNAzyme, DDZin-M = inactive anti-dengue virus DNAzyme. **B)** Analysis of DDZ-AuNP sensitivity by UV/Vis spectrophotometry. Ten microliters (10 uL) of cell culture fluid from Mock, DENV-2 NGC (titers of 1 × 10^1^, 1 × 10^2^ 1 × 10^6^/mL), or CHIKV vaccine strain 181/25 (1 × 10^6/^mL) infected C6/36 cells, or DENV serially diluted from 1 × 10^1^ (Dil1 through Dil5) or were added to separate mixtures as described in **A)**. UV/Vis spectrophotometric analysis was performed using the ND-1000 spectrophotometer at an absorbance of 520 nm. Absorbance measurements were graphed in log scale to illustrate sensitivity and accuracy of the colorimetric DENV detection method.

The sensitivity of our DENV detection system was assessed using standardized titers of DENV-2 (Figure 8). Titers of 10^1^, 10^2^,10^4^ and 10^6^ viruses/ml, as determined by TCID_50_-IFA (data not shown), were assayed using our colorimetric DDZ-M-AuNP detection method as described above. The negative controls consisted of the same reaction mixture as the experimentals lacking DENV-2 (mock), or with the catalytically inactive DDZin-M substituted for DDZ-M. Following the addition of 1.5 M NaCl and incubation at 37°C for 5 minutes samples were analyzed by visual inspection.

Positive DENV-2 detection was evident after only 5 minutes at 37°C, and demonstrated as little as 10^1^ DENV/ml could cause a color transition, although the samples containing 10^1^ and 10^2^ did not transition to a very pale purple rather than completely clear. In addition, we calculated the amount of DENV RNA corresponds to approximately 0.6 μM (for 10^6^/ml), 6 nm (for 10^4^/ml), 0.6nM (for 10^2^/ml), or 0.06 nM (for 10^1^/ml) of DENV RNA per reaction.

Further assessment of the sensitivity of our colorimetric DENV detection assay was further assessed by UV/Vis spectrophotometry using standardized titers of DENV-2 (Figure 8B). Titers of 10^1^, 10^2^, and 10^6^, viruses/ml, as determined by TCID_50_-IFA (data not shown) and five serial dilutions originating from 10^1^ (Dil1 through Dil5) were assayed using our colorimetric DDZ-M-AuNP as described in methods, and analyzed by UV/Vis spectrophotometry at an absorbance of 520. Positive detection of DENV-2 was evident with each sample that contained DENV-2 RNA, as demonstrated by a decrease in A520. This result is displayed as a greater –log10(520) value than the negative control Mock or CHIKV infected samples. Logarithmic interpretation of the resulting spectrophotometric measurements was performed to derive detection assay sensitivity. A linear relationship (R^2^ = 0.92; Figure 8B) demonstrates this assay is both sensitive and accurate. Spectrophotometric results also demonstrate our colorimetric DENV detection assay possesses the sensitivity to detect the presence of the DENV genome, even in very dilute samples (Dil4) which is of no surprise since researchers have previously detected colorimetric change associated with AuNP aggregation, by spectrophotometry, in samples containing only femtomole amounts of substrate [72,73].

## Discussion

Simple and rapid diagnostic methods to screen mosquito and patient samples for the presence of viral pathogens can significantly facilitate diagnosis and treatment of virus borne diseases in field environments where sophisticated methods of virus detection are impractical. An ideal virus detection method must distinguish the target pathogen from other diseases exhibiting similar symptoms (such as malaria, leptospirosis, typhoid, typhus and Chikungunya), be highly sensitive during the acute stage of infection, provide rapid results, be inexpensive, easy to use, and stable at temperatures greater than 30°C for use in a field environment [61]. Furthermore, DENV detection methods must show utility in epidemiological surveillance and outbreak monitoring by allowing independent detection of each serotype, and must have the ability to distinguish between primary and secondary infection [61].

In light of the caveats and pitfalls of the virus detection methods currently in use [18-24,45], the aim of this research was to explore the utility of a multiple DENV serotype targeting DNAzyme, called DDZ-M, and DENV- serotype specific DNAzymes (Table 1), coupled to AuNPs for detecting DENV. DDZ was designed to target the most conserved region of the DENV genome that includes the 5’-3’CS (Figure 2A and 2B). DENV serotype-specific DNAzymes (designated DDZ-1 through DDZ-4) were engineered to bind regions of DENV that are conserved within each serotype. The demonstrated ability of DNAzymes to successfully target small stretches of RNA makes these catalytic oligonucleotides highly useful for targeting conserved regions of virus genomes. Our results suggest that DNAzyme targeting coupled with noncrosslinking AuNP aggregation satisfies many of the criteria required to have an ideal method for DENV detection.

While our DDZ-AuNP colorimetric detection system demonstrates the capacity to target the highly conserved DENV 5’ CS region, the utility of these molecules as detection agents requires a minimal subset of anti-DENV DNAzymes (DDZs) to be occupied for aggregation of AuNPs to occur. The high tolerance of DNAzymes to mismatched binding of the target oligonucleotides [74,75] makes DNAzymes ideal for detection of viruses because they will be able to detect many closely related variants. Prior studies have demonstrated aptazymes can detect synthetically produced segments of virus genomes [45]. We have demonstrated that under optimal reaction conditions the full length genome of DENV-2 can also be detected through the aggregation of DDZ-tethered AuNPs following the interaction of the DDZ component with the DENV-2 RNA genome.

Our anti-DENV DNAzyme (DDZ), when conjugated with AuNPs, readily detects its cognate target sequence within a synthetic 170 base segment of the DENV-2 NGC RNA corresponding to the 5’UTR, 5’CS and the 5’ 74 bases of the capsid open reading frame (Figure 4). Aggregation events result from deshielding AuNPs from sodium ions following DDZ binding to the synthesized DENV-2 target [76]. The DDZ-AuNP conjugate also detects purified viral RNAs or genomic RNA liberated from cell culture derived DENV-2 NGC virions. In our analyses we utilized cell culture supernatants instead of patient blood sample or infected mosquitoes because it is more convenient to determine optimal experimentation parameters (e.g. SDS and NaCl concentrations (Figure 5A and 5B, respectively) and limits of detection (Figure 8) using a less complex cell culture system. These results provide the first confirmation of effective DENV detection using our DDZ-AuNP assay, and represent for the first time a catalytic nucleotide-based method can be used to detect DENV in fluid. Subsequent analyses will be required to optimize procedures for applications with infected patient serum or mosquito tissues.

Previous studies using oligonucleotide-tethered AuNPs have determined optimal aggregation occurs with NaCl concentrations from 1.0 M to 1.5 M, while concentrations ≥ 2.0 M destabilized conjugated AuNPs [30]. In our hands, a NaCl concentration of 1.5 M allows full aggregation of DDZ-AuNP in the presence of 0.6 μM DENV-2 RNA (Figure 5A). We may infer that the color transition observed in samples containing DENV was not due to DNAzyme activity against the AuNP or non-specific interaction with cell derived oligonucleotides since the control solution containing 0 M NaCl did not yield a false positive result.

Furthermore, DDZ-AuNP aggregation in our DENV detection assays was not driven by the loss of AuNP stability in the presence of 10 mM MgCl_2_ (Figure 6). This was not a surprising result since resistance of DNA-probe-tethered AuNPs to MgCl_2_ concentrations ≤ 10 mM have been reported [30,51].

Sodium dodecyl sulfate (SDS) proved to be an effective, low cost, detergent for directly lysing virus particles in our assay [62]. SDS titration experiments on cell culture fluids containing DENV-2 NGC (Figure 5A). demonstrated a concentration of 0.5% (w/v) was sufficient to completely lyse DENV-2 particles without interfering with AuNP aggregation reactions. Addition of this detergent to the assay components has no effect on the cleavage or aggregation reactions.

Our DDZ-AuNP colorimetric assay is capable of distinguishing between DENV-2 NGC and CHIKV (Figure 7), two symptomatically related viral pathogens, and indicates the utility of this detection approach in regions of the world that are endemic to both DENV and CHIKV [63-65]. This increases the attractiveness and utility of the assay in epidemiological surveillance of dengue viruses in regions that are endemic to multiple pathogens that display similar symptoms. UV/Vis spectrophotometric analysis of these samples showed a fifty fold decrease in absorbance at 520 nm in the presence of DENV, demonstrating our DENV detection method has the sensitivity required for use with a spectrophotometer.

This DDZ-AuNP system allows for visual detection of DENV at titers as low as 10^1^/mL, which translates to a concentration of 0.06nM DENV RNA (Figure 8A). This compares quite favorably to a previously reported RNA aptazyme-AuNP system that exhibits a sensitivity of 7.5nM [62]. Further assessment of the limits of DENV detection by UV/Vis spectrophotometric analysis (Figure 8B) demonstrates this assay displays sensitivity that is consistent with previous reports of RNA detection at sub-femtomole levels using gold nanoparticle detection systems [72,73]. Though detection of DENV RNAs at this low concentration is not physiologically relevant to what is present in posquitoes or humans, our ability to detect al this level demonstrates the power of AuNPs in detection schemes.

Moreover, despite the fact that we are detecting 1 × 10^6^ TCID50 units, there are substantially more inactive virus particles present in a given sample [77,78]. Therefore, by adding SDS to lyse DENV particles the sensitivity of our DDZ-AuNP detection method is enhanced for real world applications. DENV infected patients exhibit titers of 10^7^ to 10^8.5^ TCID_50_ units [79]. Since we can detect approximately 6 to 7 orders of magnitude or more below this, our assay could potentially allow detection of DENV in infected patients prior to the manifestation of symptoms. Current DENV detection methods lack consistent bedside detection of DENV prior to the manifestation of symptoms [80], a caveat of NS-1 antigen detection methods [81]. Secondly, individual *Ae. aegypti* mosquitoes are typically infected at a titer of 10^1^ to 10^2^ TCID_50_ units [82], well within the limits of detection for this assay, making it potentially ideal for surveillance of DENV in mosquito populations.

We have to date demonstrated that our multi-DENV serotype detecting DDZ-M-AuNP device can detect all four DENV serotypes directly from cell culture fluid without sample processing (Figure 7E). Furthermore, serotype-specific DDZ-tethered AuNPs have demonstrated utility in detecting each of the corresponding four DENV serotypes in a serotype specific manner (Figure 7E). For example, DDZ-1 tethered AuNPs detected the presence of DENV-1, and only the DENV-1 serotype, due to the designed specificity of the DDZ-1 DNAzyme to a region in the DENV-1 Capsid gene that is fully conserved solely among the DENV-1 serotype. The other serotype specific DDZ-tethered AuNPs possess this same feature in the detection of their corresponding DENV serotype (Figure 7E, see results summarized in Table 1). Full development of this system will provide a valuable method for the detection of DENV in a serotype specific manner in mosquito populations leading to enhanced speed and accuracy of epidemiological surveillance.

The DDZ-AuNP assay’s simplicity provides distinct advantages over other virus detection methods. The assay can be packaged as a pre-mixed reaction solution in eppendorf tubes, and may be performed without any specialized equipment or training. Furthermore, this assay is inexpensive, costing about $0.80 per sample, as compared to serological testing or PCR-based methods which can cost $2 per sample or more to perform. Assay components are stable for months at room temperature [83], and exhibit stability at temperatures greater than 30°C.

We anticipate further development of this assay will enable sensitive detection and discrimination of individual DENV serotypes in mosquito populations and patient derived samples as well as other virus derived RNAs. Detection prior to the onset of symptoms could allow more effective diagnosis and treatment of infected patients, and more rapid recovery from the disease. The simplicity of the assay makes it ideal as a means of early surveillance to target locations for more effective mosquito suppression strategies.

## Conclusions

The results presented here show that the DDZ-M-AuNP, designed to be active against all forms of dengue virus, is capable of effectively detecting the DENV 2-NGC genome in a sequence specific manner. Serotype specific DNAzymes tethered to AuNPs demonstrate utility in the independent identification of DENV serotypes. Coupling DNAzyme catalysis with gold nanoparticle aggregation provides an attractive alternative to other DENV detection approaches for the identification of DENV in transformed mosquito cells and tissues.

## Methods

### DNAzyme, RNAprobe and AuNP

Thiol-modified and unmodified DNAzymes were synthesized and desalted by Life Science Technologies (Grand Island, NY, USA). The oligoribonucleotide target was synthesized and HPLC-purified by Life Science Technologies. Quantification of these oligonucleotides was performed with the ND-1000 spectrophotometer from NanoDrop (Wilmington, DE). Gold colloidal solutions containing 1.6 × 10^12^ particles/mL gold nanoparticles (AuNPs) with a diameter of 15 nm were purchased from Cytodiagnostics (Burlington, ON, CA).

### Cells, virus and antibody

*Ae. albopictus* C6/36 cells were obtained from ATCC, and maintained in Leibovitz’s L-15 media (Atlanta Biologicals) supplemented with 10% FBS (Atlanta Biologicals), 10% TPB (triptose phosphate broth; Invitrogen/Gibco), penicillin G (100 U/ml; Invitrogen/Gibco) and streptomycin (100 U/ml; Invitrogen/Gibco). The C6/36 cells used in this study were maintained in a 28°C incubator and passaged every 4 days. Viral stocks were prepared as previously described [78].

The DENV strains and Genbank GenInfo identifiers for the four serotypes used in this study are as follows: DENV type 1 Hawaii: DQ672564.1, DENV type 2 strain New Guinea C (NGC): AF038403.1, DENV type 3 strain ThD3 0010 87(strain H87): AY676352.1, DENV 4 strain DENV-4/SG/06K2270DK1/2005 (strain H241): GQ398256.1.

### Design of the anti-DENV DNAzyme (DDZ) and catalytically inactive form (DDZin)

DENV sequence data was obtained from the national center of biotechnology information (NCBI). Sequences representative of all four serotypes of dengue were aligned using ClustalX [54,55]. The aligned sequences comprise the following genbank GenInfo identifiers: 12018173, 12018169, 12018171, 12659201, 2909798, 2909788, 2909786, 2909796, 6841603, 6841595, 6841605, 6841591, 6841601, 6841597, 6841593, 6841599, 6841587, 6841585, 6841589, 1000740, 1000738, 2909784, 1000736, 4926937, 4926935, 4926927, 4926929, 4926931, 2909794, 2909792, 1000742, 4926933, 2155257, 2723944, 323447, 6581076, 6581078, 2723942, 323449, 323650, 18644123, 1864412, 11119731, 19744844, 18644125, 18644127, 18643733, 4337012, 13386495, 1881708, 19071809, 13926152, 9280544, 14585842, 4926947, 4926939, 323654, 4926945, 4926943, 7329983, 7329981, 13540386, 14328931, 14485523, 323660, 17129645, 22901065, 22901063, 22901061, 1854040, 1854038, 1854036, 17129647, 24417519, 24417517, 24417515, 27656962, 24417513, 19071807, 14195698, 8927332, 14328929, 12711599, 323468, 25992053, 25992047, 25992041, 25992029, 25992025, 25992055, 25992033, 19071811, 25992043, 25992039, 25992037, 25992051, 25992031, and 25992057.

The 5’ arms of DDZ-M and DDZin-M (Table 2) were designed to bind to nucleotides 150 to 158 of the DENV genome. The 3’ arms were designed to bind to the 5’ end of the target region of the DENV genome that corresponds to nucleotides 140 to 148. These 5’ and 3’ arms of facilitated DDZ cleavage of the substrate DENV RNA between the purine-pyrimidine dinucleotide motifs G149 and C150.

**Table 2:** Nucleotide sequences of active and negative control DNAzymes and corresponding targets

**DNAzyme**	**5’Arm (5’-- > 3’)**	**3’Arm (5’-- > 3’)**	**Catalytic core**	**Target**
DDZ-M	TTTCTCTCG	GTTTCAGCA	GGCTAGCTACAACGA	TGCTGAAACGCGAGAGAAA
DDZ-1	ATCGCTCCA	TCTTCTTGA	GGCTAGCTACAACGA	TCAAGAAGAATGGAGCGAT
DDZ-2	AAAGGCGTA	TTCTCGCCT	GGCTAGCTACAACGA	AGGCGAGAAATACGCCTTT
DDZ-3	TAGCCAAGA	TCCTGCTGT	GGCTAGCTACAACGA	ACAGCAGGAGTCTTGGCTA
DDZ-4	GTTGGTTCA	TTTTCCAGA	GGCTAGCTACAACGA	TCTGGAAAAATGAACCAAC
*Negative Control*				
DDZin-M	TTTCTCTCG	GTTTCAGCA	AGCAACATCGATCGG	TGCTGAAACGCGAGAAA

Left column lists the active (DDZ-M) and inactive (DDZin-M) DNAzymes used in this report. The second and thrid columns list the sequences of the 5’and 3’ binding arms of the catalytically active DNAzymes and the inactive DDZin-M, respectively. Also shown are the sequences of the catalytic cores of each DNAzyme. The right column lists the nucleotide sequence each binding arm binds to where applicable. All sequences are displayed in a 5’ to 3’ direction. See Methods for description of DNAzyme design.

The DDZ target site was selected by scanning the 5’CS domain for one of the purine-pyrimidine dinucleotide motifs required for DNAZyme catalysis [36]. The primary criterion for selection was that a purine-pyrimidine motif located within the target site must be present in all strains of a given DENV serotype. Another important criterion for selecting suitable sites for DDZ cleavage was that the length of conserved flanking arms be long enough to insure specificity of the DNAzyme for the target site. The 5’ and 3’ arms of each DDZ were 9 bases in length since this was determined to be optimal for DNAzyme catalysis and provides a sufficient level of specificity to insure minimal off-target effects [36].

### Preparation of DDZ-tethered AuNP (DDZ-AuNP)

Preparation of DDZ-M-AuNP was performed as previously described [83] with a few modifications. The DTT-reduced thiol-DDZ-M 5’-SH-(CH2)6-d(TTTCTCTCGGGCTAGCTACAACGAGTTTCAGCA)-3’ (SH-DDZ-M) was purified by ethanol precipitation. 3 ml of AuNP and 5 mM acetate buffer (pH 5.2) were transferred to a glass scintillation vial, capped and incubated for 24 hours at room temperature. Following incubation 5 mM Tris acetate (pH 8.2) buffer and 100 mM NaCl were added and the resulting mixture was incubated at room temperature for an additional 24 hours. These functionalized particles (500 μl) were transferred into 1.7-ml microcentrifuge tubes and centrifuged at 16,110 *g* at room temperature for 15 min to remove unreacted SH-DDZ-M. The nanoparticles were redispersed in 1 mL of buffer containing 100 mM NaCl, 25 mM Tris acetate, (pH 8.2) and 0.01% SDS, centrifuged again at 16,110 g at room temperature for 15 min. The supernatant was removed and the nanoparticles were dispersed in 500 μl of buffer containing 300 mM NaCl and 25 mM Tris acetate (pH 8.2), and re-centrifuged for 15 min to remove the remaining unreacted SH-DDZ-M. The cleaned DDZ-M-AuNP were redispersed into 200 μL of buffer containing 100 mM NaCl, 25 mM Tris acetate, (pH 8.2) and 0.05% SDS. This same procedure was followed for the coupling of DENV serotype-specific DTT-reduced DNAzymes: thiol-DDZ-1 5’-SH-(CH2)6-d(AGCCAxAAAGGCTAGxCTACAACGATCCTGCTG)-3’(SH-DDZ-1), thiol-DDZ-2 5’-SH-(CH2)6- d(AAGGCGTAGGCTAGCTACAACGATTCTCGCC)-3’ (SH-DDZ-2), thiol-DDZ-3 5’-SH-(CH2)6-d(AGCCAAGAGGCTAGCTACAACGATCCTGCTG)-3’ (SH-DDZ-3), and thiol DDZ-4 5’-SH-(CH2)6-d(TTGGTTCGGCTAGCTACAACGATTTTCCAG)-3’ (SH-DDZ-4).

### Analysis of DDZ-tethered AuNPs in detecting a synthetic DENV-2 artificial target

DDZ-AuNPs (1 × 10^5^/mL) were combined in a 1.5 mL eppendorff tube with 10 mM MgCl_2_ for optimal DNAzyme activity [36], 1.0 M NaCl to drive aggregation of AuNPs [30,51,58], and synthetic DENV-2 RNA target (7.5 nM) corresponding to the 5’ 170 nucleotides of the virus genome was added. Reaction mixes were incubated at 37°C and inspected every 5 minutes over a 30 minute period. Photographs were taken with a Nikon CoolPix S3300 camera (Nikon USA, Melville, NY).

### Measurement of Mg^2+^ resistance of oligonucleotide-tethered AuNPs

This analysis was performed as previously described [30]. A mixture composed of 1 μL of DDZ-tethered AuNPs, 50 mM Tris–HCl (pH 7.5), and increasing concentrations of MgCl_2_ (5 mM to 20 mM) 10 μL were incubated at room temperature for 0 ~ 30 min. Photos of these AuNPs at each incubation time were taken with a Nikon CoolPix S3300 camera, and the absorbances were measured with a ND-1000 spectrophotometer.

### *In vitro* analysis of DDZ-tethered AuNPs

DENV RNA was isolated from DENV infected *Ae. albopictus* C6/36 cells using the QiaAmp viral RNA Mini Kit (Qiagen) according to the manufacturer’s protocol. 10 μM of eluted DENV RNA was incubated with 1 × 10^5^ DDZ-AuNP/ml for 30 min at 37°C. 15 ul of this reaction mixture was added to a RT-PCR mix (Life Science Technologies) containing heterologous and random hexametric primers to amplify the digested fragments. These RT-PCR fragments were then separated on 1.75% agarose gels.

### Sodium dodecyl sulfate (SDS) titration analysis

Ten microliters (10 μl) of cell suspension containing 1 × 10^6^ DENV-2 NGC/mL, as determined by TCID_50_-IFA, was added to a mixture containing 150 mM Tris–HCl (pH 7.5), 10 mM 10 mM MgCl_2_, 1 × 10^5^ DDZ-AuNP particles/mL, 1.5 M NaCl and SDS at concentrations ranging from 0% to 1% (w/v). Samples were incubated at 37°C for 30 minutes and analyzed every 5 min by visual inspection for aggregation of AuNPs, an indicator of positive detection of in cell culture DENV-2. Photographs were taken with a Nikon CoolPix S3300 camera.

### NaCl titration assay

DENV -2 NGC RNA were isolated from *Aedes albopictus* C6/36 cells using the Qiamp Viral RNA mini kit, and added at a concentration of 0.6 μM (~10μL) to a reaction mixture containing 150 mM Tris–HCl (pH 7.5), 10 mM 10 mM MgCl_2_, 1 × 10^5^ DDZ-AuNP particles/mL, 0.5% (w/v) SDS, and NaCl (0 M to 2 M). Mixes were incubated at 37°C for 30 minutes and analyzed every 5 min by visual inspection for aggregation of AuNPs. Samples were analyzed by visual inspection, and photographs taken. Positive detection of DENV-2 NGC RNAs was evident with a complete red to clear color transition occurring with the addition of 1.5 M NaCl.

### Determination of DDZ-AuNP specificity

Ten microliters (10 uL) of cell culture fluid containing 1 × 10^6^/mL DENV-2 NGC or, as a negative control, CHIKV vaccine strain 181/25 [84] was added to a mixture containing 150 mM Tris–HCl (pH 7.5), 10 mM 10 mM MgCl_2_, 1 × 10^5^ DDZ-M-AuNP,DDZin-M-AuNP or any of the serotype-specific DDZ tethered AuNPs/mL, 0.5% (w/v) SDS, and 1.5 M NaCl. Samples were mixed and incubated at 37°C for 5 minutes, photographs were taken using the Nikon CoolPix S3300 camera, and spectrophotometric analysis was performed using the ND-1000 spectrophotometer.

### Analysis of DDZ-AuNP limits of DENV detection

DENV-2 NGC of the titers indicated (Figure 8) were produced as follows. A titer of 1 × 10^6^/mL was obtained following inoculation of *Ae. albopictus* C6/36 cells with 0.1 MOI and incubated at 28°C for 6dpi. DENV-2 NGC were grown to titers of 1 × 10^4^/mL and 1 × 10^2^/mL at 3dpi and 6dpi, respectively, following inoculation of Vero cells with MOI of 0.1. DENV-2 NGC at a titer of 1 × 10^1^/mL were produced by serial dilution of the 1 × 10^2^/mL stock. Titers were determined by TCID_50_-IFA as described [53,66].

The DENV-2 NGC titers described above served as substrates for DDZ-AuNP colorimetric assays to determine their limits of DENV detection. Ten microliters (10 μl) of each dilution stock was added to a buffered reaction mix containing 150 mM Tris–HCl (pH 7.5), 10 mM 10 mM MgCl_2_, 1 × 10^5^ DDZ-M-AuNP particles/mL, 1.5 M NaCl, and 0.5% (w/v) SDS. Samples were mixed and incubated at 37°C for 5 minutes and photographs were taken Nikon CoolPix S3300 camera. UV/Vis spectrophotometric analysis was performed using the ND-1000 spectrophotometer.

## Competing interests

The coupled DNAzyme-gold nanoparticle detection technology is part of a pending patent application, numbers USSN 16/835,173 and USSN 61/835,758.

## Authors’ contributions

JRC engineered the DENV specific DNAzyme tethered AuNP approach and performed all RT-PCR and detection assays. VB performed all TCID50 analysis of DENV and CHIKV virus stocks to determine titer and confirm identity. CAK maintained all virus stocks, and TSF maintained all cell cultures used in this research. MJF provided technical assistance. The manuscript was prepared by JRC and MJF. All authors read and approved the final manuscript.
